# Genotype comparison of *Plasmodium vivax* and *Plasmodium falciparum* clones from pregnant and non-pregnant populations in North-west Colombia

**DOI:** 10.1186/1475-2875-11-392

**Published:** 2012-11-26

**Authors:** Eliana M Arango, Roshini Samuel, Olga M Agudelo, Jaime Carmona-Fonseca, Amanda Maestre, Stephanie K Yanow

**Affiliations:** 1 Grupo Salud y Comunidad, Facultad de Medicina, Universidad de Antioquia, Medellín, Colombia; 2 Provincial Laboratory for Public Health, Edmonton, Canada; 3 School of Public Health, University of Alberta, Edmonton, Canada

**Keywords:** Malaria, Pregnancy, Colombia, *P. falciparum*, *P. vivax*, Placenta, Genotyping, Genetic diversity, Genetic differentiation

## Abstract

**Background:**

Placental malaria is the predominant pathology secondary to malaria in pregnancy, causing substantial maternal and infant morbidity and mortality in tropical areas. While it is clear that placental parasites are phenotypically different from those in the peripheral circulation, it is not known whether unique genotypes are associated specifically with placental infection or perhaps more generally with pregnancy. In this study, genetic analysis was performed on *Plasmodium vivax* and *Plasmodium falciparum* parasites isolated from peripheral and placental blood in pregnant women living in North-west Colombia, and compared with parasites causing acute malaria in non-pregnant populations.

**Methods:**

A total of 57 pregnant women at delivery with malaria infection confirmed by real-time PCR in peripheral or placental blood were included, as well as 50 pregnant women in antenatal care and 80 men or non-pregnant women with acute malaria confirmed by a positive thick smear for *P. vivax* or *P. falciparum*. Five molecular markers per species were genotyped by nested PCR and capillary electrophoresis. Genetic diversity and the fixation index F_ST_ per species and study group were calculated and compared.

**Results:**

Almost all infections at delivery were asymptomatic with significantly lower levels of infection compared with the groups with acute malaria. Expected heterozygosity for *P. vivax* molecular markers ranged from 0.765 to 0.928 and for *P. falciparum* markers ranged from 0.331 to 0.604. For *P. vivax* infections, the genetic diversity was similar amongst the four study groups and the fixation index from each pairwise comparison failed to show significant genetic differentiation. For *P. falciparum*, no genetic differentiation was observed between placental and peripheral parasites from the same woman at delivery, but the parasites isolated at delivery showed significant genetic differentiation compared with parasites isolated from subjects with acute malaria.

**Conclusions:**

In North-west Colombia, *P. vivax* parasites have high genetic diversity that is equivalent in pregnant and non-pregnant populations as well as in symptomatic and asymptomatic infections. For *P. falciparum*, the overall genetic diversity is lower, with specific genotypes associated with asymptomatic infections at delivery.

## Background

Malaria in pregnancy causes substantial maternal and infant morbidity and mortality in tropical areas due to increased risk of adverse pregnancy outcomes such as miscarriage, maternal anaemia and low infant birth weight [1]. Both *Plasmodium falciparum* and *Plasmodium vivax* infections cause adverse pregnancy outcomes, however only *P. falciparum* has been studied extensively [2-5]. The predominant pathology secondary to maternal *P. falciparum* infection is placental malaria. It is known that *P. falciparum*–infected red blood cells (Pf-iRBCs) accumulate in the placenta by binding to chondroitin sulfate A (CSA) through the ligand VAR2CSA, a 350-kD member of the *P. falciparum* erythrocyte membrane protein 1 (PfEMP1) family of surface adhesion antigens [3,6]. PfEMP1 proteins are encoded by ~60 members of the *var* multigene family and their expression appears to be controlled by allelic exclusion, whereby each parasite expresses a single variant on the surface of the iRBC. Antigenic variation is induced by switching expression of alternative PfEMP1 variants [7]. In pregnancy, the expression of different adhesion molecules can result in selection and segregation of parasite subpopulations in the peripheral and placental circulation [8]. Pf-iRBCs isolated from placenta bind to CSA but not to CD36, the primary receptor mediating Pf-iRBC binding to the microvasculature [3,6,8]. *Plasmodium vivax*–iRBCs also adhere to immobilized CSA and to fresh placental cells *in vitro*, but do not adhere to immobilized CD36 [9,10]. However, the identity of the parasite ligand that mediates binding to CSA is unknown.

While it is clear that placental parasites are phenotypically different from those in the peripheral circulation, it is not known whether they also differ genetically. In several African studies, genotyping of matched peripheral and placental *P. falciparum* parasites from parturient women using the *msp1 *and/or *msp2* genes showed that the majority of alleles were shared, yet some alleles were detected exclusively in one compartment [11-16]. In Latin America, it is not known whether plasmodial parasites in pregnancy have similar genetic characteristics to those in Africa; specifically, it is not known whether parasites in the placenta and peripheral blood of pregnant women are genetically related, or whether certain genotypes are specific to infections in pregnancy. To address these questions, genetic analysis was carried out of parasites from different population groups resident in the Urabá-Altos Sinú-San Jorge-Bajo Cauca region in North-west Colombia which accounts for 60% of all cases in the country [17]. Both *P. falciparum* and *P. vivax* cause gestational and placental malaria in this region [18-21]. Genotypes of *P. vivax* and *P. falciparum* parasites isolated from peripheral and placental blood in pregnant women were compared with strains causing acute malaria in non-pregnant populations resident in the same region.

## Methods

### Study population and design

Patients recruited in this study resided in the municipalities of Turbo (08°05^′^N,76°44^′^W) and Necoclí (08°25^′^N,76°47^′^W) of the Antioquia department, and Puerto Libertador (07°54^′^N,75°40^′^W) of the Córdoba department. Together, these departments comprise a high malaria transmission area of Colombia, termed Urabá-Altos Sinú-San Jorge-Bajo Cauca. This region has an estimated area of 43506 km^2^, with 35 municipalities and a population of 2.5 million at risk of malaria. The epidemiological characteristics of this region have been described elsewhere [17,22,23]. The mean annual parasitic index (malaria cases/1000 inhabitants) during 2000–2009 was 46.6 in Turbo, 74.4 in Necoclí, and 23.4 in Puerto Libertador. *Plasmodium vivax* was reported in about 70% of malaria cases in the region by microscopic diagnosis [24,25].

This study included 57 pregnant women at delivery (“delivery group”) with a malaria infection confirmed by quantitative real-time PCR (qPCR) in peripheral and/or placental blood. For comparison, another group of 50 pregnant women with a positive thick smear for *P. vivax* (n = 30) or *P. falciparum* (n = 20) during antenatal care (“antenatal group”) were included. A third group included 80 men or non-pregnant women (“non-pregnant group”) with a positive thick smear for *P. vivax* (n = 20 men and 20 women) or *P. falciparum* (n = 20 men and 20 women). All subjects resided in the same three municipalities.

### Inclusion and exclusion criteria

The general inclusion criteria for the study population were permanent residency in a malaria-endemic community of Turbo, Necoclí or Puerto Libertador, absence of serious general disease, complicated pregnancy or complicated malaria, and informed consent. The only exclusion criterion was consent withdrawal.

### Specimen collection

Blood samples in the delivery group were collected in EDTA tubes within 8 hours of delivery. Maternal peripheral blood (delivery-periphery group) was obtained by venipuncture. Placental blood (delivery-placenta group) was collected from a pool formed on the maternal side when small sections of placenta were removed (approximate 1 cm^3^) after cleaning with saline. Peripheral blood from subjects in groups “antenatal” and “non-pregnant” was collected by venipuncture, prior to antimalarial treatment.

Thick smears were made for microscopic examination and dried blood spots were prepared on filter paper (Whatman 3MM) for DNA extraction. Blood spots were sealed in plastic bags, stored at 4°C and transported to the laboratory in Medellín.

### Malaria diagnostic tests

Field-stained thick films were read by an experienced microscopist in the local research laboratory. Parasite density was measured by counting the number of parasites per 200 leukocytes, based on a mean count of 8,000 leukocytes/μL of blood. All subjects with a peripheral blood thick smear positive for malaria received anti-malarial treatment according to the guidelines of the Colombian health authorities [26].

For diagnosis by qPCR, an alcohol-sterilized hole punch was used to cut a circle (approximately 6 mm) from each filter paper and DNA was extracted using the Saponin-Chelex method described by Plowe *et al.* 1995 [27]. The qPCR was performed as described by Shokoples *et al.* 2009 [28]. Samples were first tested for *Plasmodium* using a genus-specific set of primers and hydrolysis probe (Plasprobe). Real-time PCR was performed on the ABI 7500 FAST platform, under universal cycling conditions. Samples with a Cycle Threshold (Ct) value under 45 were tested in a duplex species-specific real-time PCR reaction for *P. falciparum* and *P. vivax*[28]. Parasite DNA concentration was quantified in the genus-specific screening reaction against a plasmid standard curve of known copy number included in each run. Concentrations are reported as the number of copies of the 18*S* rRNA gene per microliter of purified DNA.

### Molecular genotyping

All molecular markers were amplified by nested or semi-nested PCR, using 3 μL of extracted DNA as template in the first amplification step and 1 μL of the first PCR product for the second amplification. For samples positive for *P. vivax*, the microsatellites 1.501, 3.502, 3.27, and MS16, as well as the *msp3α* gene were analyzed based on the protocol described by Koepfli *et al.* 2009 [29]. The PCR reaction was performed in a final volume of 20 μL containing 1× PCR buffer (Qiagen), 2 mM of MgCL_2_ (Qiagen), 200 μM of each dNTP (Takara Bio), 0.25 μM of each primer, and 1.5 units of HotStar Taq DNA polymerase (Qiagen).The cycling program was as follows: initial denaturation for 5 min at 95°C; 30 cycles of 1 min at 95°C, 1 min at 56°C–60°C (according to the marker analysed [29]), 1 min at 72°C; and a final step for 5 min at 72°C.

Samples positive for *P. falciparum* were genotyped based on the microsatellites ARA2, TA1, Polyα and PFPK2 and the gene *msp2*. Amplification of microsatellites was according to the protocol described by Anderson *et al.*[30]. The reaction volume was 15 μL containing 1× PCR buffer, 2 mM of MgCL_2_, 200 μM of each dNTP, 0.25 μM of each primer, and 0.2 units of HotStar Taq DNA polymerase. The cycling conditions were as follows: initial denaturation for 2 min at 94°C; 45 cycles of 30 sec at 94°C, 30 sec at 42°C, 30 sec at 40°C, and 40 sec at 65°C for the first reaction. The nested reaction consisted of 40 cycles of: 30 sec at 94°C, 30 sec at 45°C and 30 sec at 65°C. The final elongation step in both reactions was 5 min at 65°C. The *msp2* gene was amplified based on the protocol described by Felger and Beck [31]. The reaction volume was 20 μL containing 1× PCR buffer, 1.5 mM of MgCL_2_, 200 μM of each dNTP, 0.25 μM of each primer for the first reaction and 0.4 μM for the nested reaction, and 0.6 units of HotStar Taq DNA polymerase. The cycling program was: initial denaturation for 2.5 min at 94°C; 40 cycles of 30 sec at 94°C, 45 sec at 42°C for the first reaction or 50°C for the nested reaction, and elongation for 1.5 min at 70°C with a final elongation step of 10 min at 70°C.

All PCR analyses were performed in an Applied Biosystems 2720 Thermal Cycler. Amplification was confirmed by visualization in 2% agarose gels and PCR products were stored at 4°C in the dark. Product sizes were resolved by capillary electrophoresis in an ABI Prism 3100 Genetic Analyzer (Perkin Elmer Applied Biosystems), using GS500 LIZ as internal size standard and the microsatellite conditions as the default settings. The results were analysed using GeneMapper software (version 3.5; Applied Biosystems). All electropherograms were visually inspected; peaks above a cut-off of 300 relative fluorescent units (RFU) were considered true amplification products. Alleles were grouped manually based on their repeat length: 3-bp bins for all the *P. falciparum* microsatellites, PvMS16 and *Pvmsp3α*; 4-bp bins for Pv3.27; 7-bp bins for Pv1.501 or 8-bp bins for Pv3.502. Multiple alleles per locus were scored if minor peaks were >33% of the height of the predominant allele present for each locus. All mixed infections were genotyped with molecular makers of both species.

### Statistical analysis

Microsatellite analyzer version 4.05 [32] was used for calculating allele frequency, expected heterozygosity (*He*) and the F_ST_ index. *He* was defined as the probability that two clones selected from the population at random carry different alleles, and was calculated with the formula *He* = [n/(n − 1)] [1 − *Σ*p_i_^2^, where n is the number of isolates analyzed and p_i_ is the frequency of the i^th^ allele in the population. The F_ST_ index [33] with pairwise comparisons was used to evaluate the genetic differentiation between subpopulations of parasites isolated from the different groups of patients (delivery-periphery vs. delivery-placenta vs. antenatal vs. non-pregnant). Each F_ST_ value was tested to determine whether it was statistically different from 0, involving 10000 random permutations of the data. Kruskal-Wallis and Chi-squared tests were used for comparison of continuous and categorical variables, respectively. Significance was set at p <0.05.

### Ethical considerations

Patients or guardians, in case of <18 years of age, signed a voluntary consent form. The study involved minor risk and approval was granted by the Comité de Ética of Instituto de Investigaciones Médicas, Facultad de Medicina, Universidad de Antioquia (Approval Certificate: IIM889ADV) and the Health Research Ethics Board of the University of Alberta.

## Results

### Malaria infection in the three study groups

Women with malaria infection at delivery (delivery group) had a mean age of 23 ± 6 years (range of 14 to 38 years) and 31% were primiparous. Of the 57 women in this group, 31 (54%) had a plasmodial infection detected by qPCR in both peripheral and placental blood, 17 (30%) had infection only in placental blood, and 9 (16%) were only positive in peripheral blood, for a total of 88 infections at delivery. *P. falciparum* was detected in 47% (41/88) of infections, *P. vivax* in 43% (38/88) and 10% (9/88) were mixed infections. Almost all women in the delivery group were asymptomatic; only seven women (12%) had symptoms associated with malaria.

The mean age of pregnant women attending antenatal care (antenatal group) was 22 ± 6 years (range of 13 to 38), 16% were in their first trimester of pregnancy, 59% in the second trimester and 25% in the third. The qPCR test confirmed *P. vivax* monoinfection in all 30 samples diagnosed by thick smear, while *P. falciparum* monoinfection was confirmed in 19 out of 20 samples, with one sample diagnosed as *P. falciparum* by thick smear but with a mixed infection detected by qPCR.

The mean age in the non-pregnant population group (non-pregnant group) was higher than the other two groups (27 ± 16 years, range of 8 to 65), but statistically similar (p = 0.2509). *Plasmodium falciparum* monoinfection was confirmed by qPCR in 38 of 40 samples, *P. vivax* monoinfection in 39 of 40, and there were three mixed infections. Individuals in both antenatal and non-pregnant groups had symptomatic malaria and received the treatment recommended by the Colombia health authorities based on the thick smear result.

The infection level quantified by thick smear (parasites/μL) or qPCR (DNA copies/μL template) was significantly lower in the delivery group, particularly for *P. falciparum* infections (Table 1). The low concentration of parasite DNA influenced the success in genotyping; in the delivery group, genotyping at any marker failed in 11 out of 97 samples (two peripheral and seven placental samples), 28 samples were genotyped at one marker (12 peripheral and 16 placental) and 58 were successfully genotyped at two or more markers (28 peripheral and 30 placental). In the antenatal and non-pregnant groups, which had higher levels of parasite DNA, 88% (118/134 infections in both groups) of samples were genotyped for the five genetic markers while four markers were genotyped in the remaining samples. Overall, successful genotyping for each marker varied from 78% to 91% for *P. vivax* samples and from 62% to 79% for *P. falciparum* (Table 2).

**Table 1:** Level of plasmodial infection according to the study groups

**Study group**	***Plasmodium vivax***			***Plasmodium falciparum***		
	**n**	**Parasites/μL (mean±SD)**	**DNA copies/μL (mean±SD)**	**n**	**Parasite/μL (mean±SD)**	**DNA copies/μL (mean±SD)**
Delivery-periphery	22	960 ± 2134	382 ± 732*	21	208 ± 843	16 ± 33*
Delivery-placenta	25	89 ± 247	118 ± 234	29	5 ± 17	88 ± 288
Antenatal	31	6464 ± 6467	1825 ± 2326	20	6124 ± 8245	1222 ± 2052
Non-pregnant	42	7000 ± 7585	3020 ± 5372*	41	6852 ± 13273	1521 ± 2715*

* p < 0.05 for comparison between *P. vivax* and *P. falciparum.*

**Table 2:** Genetic diversity of each molecular marker

	***Plasmodium vivax *(n = 120)**				
	**1.501**	**3.502**	**3.27**	**MS16**	**MPS3α**
Successful genotyping (%)	91	89	84	79	78
No. different alleles detected	8	8	23	24	19
Polyclonal samples (%)	12	13	55	12	25
Expected heterozygosity (*He*)	0.765	0.836	0.894	0.928	0.881
	***Plasmodium falciparum *(n = 111)**				
	**ARA2**	**TA1**	**POLYα**	**PFPK2**	**MSP2**
Successful genotyping (%)	66	79	62	69	71
No. different alleles detected	2	4	6	6	5
Polyclonal samples (%)	3	4	7	8	9
Expected heterozygosity (*He*)	0.331	0.494	0.511	0.604	0.544

### Genetic diversity and parasite differentiation

*Plasmodium vivax* showed significantly higher genetic diversity than *P. falciparum* (Table 2). The number of different alleles detected for each marker ranged from 8 to 24 for *P. vivax* and from 2 to 6 for *P. falciparum*. Furthermore, the probability that two clones selected at random from the population carry different alleles at each marker varied from 77% to 93% for *P. vivax* and from 33% to 60% for *P. falciparum* (Table 2). Another distinction between the species is that polyclonal infections were more common in *P. vivax* than *P. falciparum* infections. Multiple alleles were detected in 62% (76/122) of *P. vivax* samples, and only 17% (19/113) of *P. falciparum* samples.

The mean *He* was similar for *P. vivax* in all study groups yet for *P. falciparum*, the delivery samples had a higher *He* than the antenatal and non-pregnant groups (Table 3). Interestingly, infections with either species at delivery showed a significantly lower frequency of multiple alleles compared with the samples from acute infection. Within the delivery group, there was no statistical difference in the *He* and the frequency of polyclonal infections between peripheral and placental samples. Likewise, there were no differences in *He* and polyclonal infections between antenatal and non-pregnant groups.

**Table 3:** Expected heterozygosity and polyclonal infections in each study group

**Study group**	***Plasmodium vivax***		
	n*	**Expected heterozygosity**	**Polyclonal infections**
		**Mean±SD**	**%**
Delivery-periphery	21	0.876 ± 0.072	47
Delivery-placenta	23	0.869 ± 0.093	33
Antenatal	31	0.847 ± 0.073	84
Non-pregnant	42	0.874 ± 0.046	73
p		0.3373	0.0004
	***Plasmodium falciparum***		
Delivery-periphery	20	0.554 ± 0.120	5
Delivery-placenta	22	0.629 ± 0.145	8
Antenatal	20	0.481 ± 0.103	25
Non-pregnant	41	0.353 ± 0.215	25
p		<0.0001	0.1462

*Number of samples successfully amplified for at least one molecular marker in each species.

Calculation of the F_ST_ index per pair of study groups (delivery-periphery vs. delivery-placenta vs. antenatal vs. non-pregnant) for *P. vivax* infections did not show significant genetic differentiation (Table 4). However, for *P. falciparum* samples, the global F_ST_ based on all loci was 0.154 (p = 0.0001) and significant differentiation was detected when parasites isolated from peripheral and placental blood of pregnant women at delivery were compared with parasites isolated from antenatal and non-pregnant subjects. The highest genetic differentiation was observed between parasites from the delivery (periphery or placenta) and non-pregnant groups. The comparison of peripheral and placental parasites within the delivery group did not display significant genetic differentiation. In addition, a comparison of the predominant alleles revealed that only one of five markers from the placental parasites were shared with those from the antenatal and non-pregnant groups, while peripheral parasites at delivery shared 3 of 5 markers (Table 5). The predominant alleles detected in both the antenatal and non-pregnant groups were exactly the same for all five markers. Moreover, of 14 women with falciparum infection in peripheral and placental blood simultaneously, 8 (57%) had the same parasite genotype in both compartments. One woman had different genotypes in these compartments and in five women it was not possible to compare the genotypes because the molecular markers failed to amplify.

**Table 4:** **Pairwise F**_**ST **_**index of *P. vivax *and *P. falciparum *isolated from each study group**

**Study group**	***Plasmodium vivax***		
	**Delivery-placenta**	**Antenatal**	**Non-pregnant**
Delivery-periphery	−0.1014	−0.0394	−0.0344
Delivery-placenta	**---**	−0.0496	−0.0464
Antenatal		**---**	−0.0229
	***Plasmodium falciparum***		
Delivery-periphery	−0.3116	0.0860*	0.2672*
Delivery-placenta	**---**	0.1020*	0.2722*
Antenatal		**---**	−0.0317

*p < 0.05.

**Table 5:** Number of alleles detected and predominant allele (frequency) of each marker per study group and species

***Plasmodium vivax***										
**Study group**	**1.501**		**3.502**		**3.27**		**MS16**		**MSP3α**	
	**alleles**	**predominant**	**alleles**	**predominant**	**alleles**	**predominant**	**alleles**	**predominant**	**alleles**	**predominant**
Delivery-periphery	5	100 bp (0.400)	8	141 bp (0.294)	12	283 bp (0.300)	11	408 bp (0.250)	11	465 bp (0.278)
Delivery-placenta	5	107 bp (0.421)	6	149 bp (0.211)	8	283 bp (0.417)	9	408 bp (0.200)	9	465 bp (0.200)
Antenatal	5	107 bp (0.389)	6	149 bp (0.257)	13	283 bp (0.222)	13	414 pb (0.267)	13	465 bp (0.303)
Non-pregnant	7	107 bp (0.244)	7	149 bp (0.261)	21	283 bp (0.242)	17	408 bp (0.145)	14	465 bp (0.271)
***Plasmodium falciparum***										
**Study group**	**ARA2**		**TA1**		**POLYα**		**PFPK2**		**MSP2**	
	**alleles**	**predominant**	**alleles**	**predominant**	**alleles**	**predominant**	**alleles**	**predominant**	**alleles**	**predominant**
Delivery-periphery	2	65 bp (0.714)	3	141 bp (0.714)	2	149 bp (0.750)	3	174 bp (0.571)	3	FC27 (0.583)
Delivery-placenta	2	65 bp (0.556)	2	141 bp (0.706)	2	149 bp (0.500)	3	171 bp (0.444)	3	3D7 (0.545)
Antenatal	2	71 bp (0.760)	4	171 bp (0.760)	3	149 bp (0.680)	5	174 bp (0.607)	3	FC27 (0.600)
Non-pregnant	2	71 bp (0.902)	3	171 bp (0.962)	6	149 bp (0.680)	4	174 bp (0.588)	5	FC27 (0.735)

## Discussion

The current study compared the genotypes of *P. vivax* and *P. falciparum* clones isolated from pregnant and non-pregnant infected subjects in North-west Colombia, using five genetic markers per species. Consistent with previous reports from non-pregnant populations, the genetic diversity of *P. vivax* is much greater than that of *P. falciparum*[34-36], which is explained by the different origins of both species and their distant phylogenetic relationship [37,38]. Other studies in Colombia have confirmed high genetic diversity for *P. vivax* infections [39-41] and low diversity for *P. falciparum*[42-45] in naturally infected, non-pregnant patients.

The number of different alleles detected and *He* were high in all *P. vivax* infections, regardless of the population (delivery, antenatal, non-pregnant) or the compartment from the same subjects (delivery-periphery, delivery-placenta). However, polyclonal infections were significantly more common in antenatal and non-pregnant than in delivery in both peripheral and placental infections. A similar finding was observed for *P. falciparum* infections; higher genetic diversity and fewer polyclonal infections were detected in the delivery group compared with the antenatal and non-pregnant groups. This can be explained by the low level of parasitaemia and parasite DNA copies detected in women of the delivery group, consistent with asymptomatic infection. Other authors also reported an association between low parasitaemia and low complexity infections [46-48].

The selection criteria used in this study likely explains the differences in parasite density observed across the study groups; all parturient women included here (delivery group) were part of a larger prevalence study on malaria infection at delivery, whose plasmodial infection was detected only by PCR (submicroscopic infection) in most cases. In contrast, selection of subjects included in the other two groups (antenatal and non-pregnant) was based on a positive thick smear (microscopic infection). Interestingly, in both groups with microscopic infection (antenatal and non-pregnant), the level of parasitaemia was similar for *P. falciparum* and *P. vivax* infections. This finding agrees with previous reports from Colombia that both *P. vivax* and *P. falciparum* uncomplicated malaria patients have low parasitaemia (<10,000 parasites/μL) [49-51]. However, reports from other endemic regions where both species co-circulate generally describe lower parasitaemias for *P. vivax* infections [5,52-54] attributed to the fact that *P. vivax* exclusively invades reticulocytes [55,56].

One of the most significant findings in this study is that *P. falciparum* infections showed specific parasite genotypes associated with delivery vs. non-pregnant populations. Two hypotheses could explain this finding. First, different parasite genotypes can be associated with low vs. high levels of parasitaemia. Previous studies in non-pregnant populations have reported association of specific genotypes with low parasitaemic and asymptomatic infections [57], as well as genotypes associated with severe malaria [58]. Second, genetically distinct subpopulations of *P. falciparum* may segregate differentially to peripheral and placental blood. Other authors have compared *P. falciparum* clones isolated from matched peripheral and placental blood using genes that encode surface proteins such as *msp1* and *msp2*[11-16] or genes associated with antimalarial drug resistance such as *dhfr*, *dhps*[59,60], and *pfcrt*[61,62]. Although the *dhfr-dhps* studies failed to find an association of specific genotypes per compartment, the *msp1-msp2* and *pfcrt* studies did identify specific genotypes present exclusively in the placenta.

The genetic diversity and differentiation of placental parasites in *P. falciparum* infections suggests that different strains or parasite genotypes share the phenotypic property of adherence to placental tissue. Genetic characterization of placental parasites should therefore be considered in the development of a pregnancy-associated malaria vaccine to ensure maximum cross-protection against genetically different strains.

## Conclusions

This study affirms that in North-west Colombia *P. vivax* parasites have high genetic diversity, which is conserved in pregnant and non-pregnant populations as well as in symptomatic and asymptomatic infections. For *P. falciparum*, the genetic diversity is lower compared with *P. vivax* with specific genotypes that are associated with low-level, asymptomatic infections at delivery.

## Abbreviations

Pf-iRBCs: *P. falciparum*–infected red blood cells; CSA: Chondroitin sulfate A; PfEMP1: *P. falciparum* erythrocyte membrane protein 1; qPCR: Quantitative real-time polymerase chain reaction; Ct: Cycle threshold; RFU: Relative fluorescence units; *He*: Expected heterozygosity; bp: Base pairs.

## Competing interests

The authors affirm that they have no commercial or other association that might pose a conflict of interest.

## Authors’ contributions

EMA participated in the study design, carried out the molecular genotyping and statistical analyses and wrote the manuscript. RS participated in the molecular genotyping. OMA participated in the collection of field samples. JCF participated in the study design, statistical analyses and preparation of the manuscript. AM and SKY participated in the study design, coordination and preparation of the manuscript. All authors read and approved the final manuscript.

## References

[B1] Desai M ter Kuile FO Nosten F McGready R Asamoa K Brabin B Newman RD Epidemiology and burden of malaria in pregnancy Lancet Infect Dis 2007 7 93 104 10.1016/S1473-3099(07)70021-X 17251080

[B2] Rijken MJ McGready R Boel ME Poespoprodjo R Singh N Syafruddin D Rogerson S Nosten F Malaria in pregnancy in the Asia-Pacific region Lancet Infect Dis 2012 12 75 88 10.1016/S1473-3099(11)70315-2 22192132

[B3] Rogerson SJ Hviid L Duffy PE Leke RF Taylor DW Malaria in pregnancy: pathogenesis and immunity Lancet Infect Dis 2007 7 105 117 10.1016/S1473-3099(07)70022-1 17251081

[B4] Hartman TK Rogerson SJ Fischer PR The impact of maternal malaria on newborns Ann Trop Paediatr 2010 30 271 282 10.1179/146532810X12858955921032 21118620

[B5] Poespoprodjo JR Fobia W Kenangalem E Lampah DA Warikar N Seal A McGready R Sugiarto P Tjitra E Anstey NM Price RN Adverse pregnancy outcomes in an area where multidrug-resistant Plasmodium vivax and Plasmodium falciparum infections are endemic Clin Infect Dis 2008 46 1374 1381 10.1086/586743 18419439 PMC2875100

[B6] Gamain B Smith JD Viebig NK Gysin J Scherf A Pregnancy-associated malaria: parasite binding, natural immunity and vaccine development Int J Parasitol 2007 37 273 283 10.1016/j.ijpara.2006.11.011 17224156

[B7] Scherf A Lopez-Rubio JJ Riviere L Antigenic variation in Plasmodium falciparum Annu Rev Microbiol 2008 62 445 470 10.1146/annurev.micro.61.080706.093134 18785843

[B8] Smith JD Deitsch KW Pregnancy-associated malaria and the prospects for syndrome-specific antimalaria vaccines J Exp Med 2004 200 1093 1097 10.1084/jem.20041974 15520241 PMC2211864

[B9] Carvalho BO Lopes SC Nogueira PA Orlandi PP Bargieri DY Blanco YC Mamoni R Leite JA Rodrigues MM Soares IS Oliveira TR Wunderlich G Lacerda MV del Portillo HA Araújo MO Russell B Suwanarusk R Snounou G Rénia L Costa FT On the cytoadhesion of Plasmodium vivax-infected erythrocytes J Infect Dis 2010 202 638 647 10.1086/654815 20617923

[B10] Chotivanich K Udomsangpetch R Suwanarusk R Pukrittayakamee S Wilairatana P Beeson JG Day NP White NJ Plasmodium vivax adherence to placental glycosaminoglycans PLoS One 2012 7 e34509 10.1371/journal.pone.0034509 22529919 PMC3328474

[B11] Kamwendo DD Dzinjalamala FK Snounou G Kanjala MC Mhango CG Molyneux ME Rogerson SJ Plasmodium falciparum: PCR detection and genotyping of isolates from peripheral, placental, and cord blood of pregnant Malawian women and their infants Trans R Soc Trop Med Hyg 2002 96 145 149 10.1016/S0035-9203(02)90284-1 12055802

[B12] Kassberger F Birkenmaier A Khattab A Kremsner PG Klinkert MQ PCR typing of Plasmodium falciparum in matched peripheral, placental and umbilical cord blood Parasitol Res 2002 88 1073 1079 10.1007/s00436-002-0715-3 12444458

[B13] Mayengue PI Rieth H Khattab A Issifou S Kremsner PG Klinkert MQ Ntoumi F Submicroscopic Plasmodium falciparum infections and multiplicity of infection in matched peripheral, placental and umbilical cord blood samples from Gabonese women Trop Med Int Health 2004 9 949 958 10.1111/j.1365-3156.2004.01294.x 15361107

[B14] Jafari-Guemouri S Ndam NT Bertin G Renart E Sow S Le Hesran JY Deloron P Demonstration of a high level of parasite population homology by quantification of Plasmodium falciparum alleles in matched peripheral, placental, and umbilical cord blood samples J Clin Microbiol 2005 43 2980 2983 10.1128/JCM.43.6.2980-2983.2005 15956437 PMC1151910

[B15] Schleiermacher D Le Hesran JY Ndiaye JL Perraut R Gaye A Mercereau-Puijalon O Hidden Plasmodium falciparum parasites in human infections: different genotype distribution in the peripheral circulation and in the placenta Infect Genet Evol 2002 2 97 105 10.1016/S1567-1348(02)00085-0 12797985

[B16] Arango E Maestre A Carmona-Fonseca J Efecto de la infección submicroscópica o policlonal de Plasmodium falciparum sobre la madre y el producto de la gestación. Revisión sistemática Rev Bras Epidemiol 2010 13 373 386 10.1590/S1415-790X2010000300002 20857025

[B17] Padilla-Rodríguez JC Álvarez-Uribe G Montoya-Araújo R Caparro-Narváez P Herrera-Valencia S Epidemiology and control of malaria in Colombia Mem Inst Oswaldo Cruz 2011 106 Suppl 1 114 122 21881765 10.1590/s0074-02762011000900015 PMC4830684

[B18] Carmona-Fonseca J Maestre A Incidencia de las malarias gestacional, congénita y placentaria en Urabá (Antioquia, Colombia), 2005–2007 Rev Colomb Obstet Ginecol 2009 60 19 33

[B19] Campos IM Uribe ML Cuesta C Franco-Gallego A Carmona-Fonseca J Maestre A Diagnosis of gestational, congenital, and placental malaria in Colombia: comparison of the efficacy of microscopy, nested polymerase chain reaction, and histopathology Am J Trop Med Hyg 2011 84 929 935 10.4269/ajtmh.2011.10-0507 21633030 PMC3110370

[B20] Padilla A Tiburcio H Apolinario M Gestación y Malaria Ginecol Obstet 1997 43 239 243

[B21] Martínez-Espinosa FE Daniel-Ribeiro CT Alecrim WD Malaria during pregnancy in a reference centre from the Brazilian Amazon: unexpected increase in the frequency of Plasmodium falciparum infections Mem Inst Oswaldo Cruz 2004 99 19 21 10.1590/S0074-02762004000100003 15057341

[B22] Carmona-Fonseca J La malaria en Colombia, Antioquia y las zonas de Urabá y Bajo Cauca: panorama para interpretar la falla terapéutica antimalárica. Parte 1 Iatreia 2003 16 299 318

[B23] Carmona-Fonseca J La malaria en Colombia, Antioquia y las zonas de Urabá y Bajo Cauca: panorama para interpretar la falla terapéutica antimalárica. Parte 2 Iatreia 2004 17 34 53

[B24] Secretaría de Desarrollo de la Salud de Córdoba-SDSC Programa Prevención y Control de Enfermedades Transmitidas por Vectores 2009 http://www.saludcordoba.gov.co/presentaciones/presentacion_etv.pptx

[B25] Dirección Seccional de Salud de Antioquia-DSSA Enfermedades Transmitidas por Vectores 2000–2010 http://www.dssa.gov.co/index.php/documentos/doc_download/669-enfermedades-transmitidas-por-vectores

[B26] Guía para la atención clínica integral del paciente con malaria http://dssa.media.vcb.com.co/dssa.gov.co/documentos/Protocolos-Vectores-INS/Clinica-Malaria.pdf

[B27] Plowe CV Djimde A Bouare M Doumbo O Wellems TE Pyrimethamine and proguanil resistance-conferring mutations in Plasmodium falciparum dihydrofolate reductase: polymerase chain reaction methods for surveillance in Africa Am J Trop Med Hyg 1995 52 565 568 7611566 10.4269/ajtmh.1995.52.565

[B28] Shokoples SE Ndao M Kowalewska-Grochowska K Yanow SK Multiplexed real-time PCR assay for discrimination of Plasmodium species with improved sensitivity for mixed infections J Clin Microbiol 2009 47 975 980 10.1128/JCM.01858-08 19244467 PMC2668309

[B29] Koepfli C Mueller I Marfurt J Goroti M Sie A Oa O Genton B Beck HP Felger I Evaluation of Plasmodium vivax genotyping markers for molecular monitoring in clinical trials J Infect Dis 2009 199 1074 1080 10.1086/597303 19275476

[B30] Anderson TJ Su XZ Bockarie M Lagog M Day KP Twelve microsatellite markers for characterization of Plasmodium falciparum from finger-prick blood samples Parasitology 1999 119 Pt 2 113 125 10466118 10.1017/s0031182099004552

[B31] Felger I Beck H Moll K, Ljungström I, Perlmann H, Scherf A, Wahlgren M msp2 genotyping of Plasmodium falciparum by capillary electrophoresis andGeneMapper® Program Methods in Malaria Research 2008 Fifth Manassas: Malaria Research and Reference Reagent Resource Center (MR4) 243 247

[B32] Dieringer D Schlötterer C Microsatellite analyser (MSA): a platform independent analysis tool for large microsatellite data sets Mol Ecol Notes 2003 3 168 169

[B33] Weir B Cockerham C Estimating F-statistics for the analysis of population structure Evolution 1984 38 1358 1370 10.2307/2408641 28563791

[B34] Ord RL Tami A Sutherland CJ ama1 genes of sympatric Plasmodium vivax and P. falciparum from Venezuela differ significantly in genetic diversity and recombination frequency PLoS One 2008 3 e3366 10.1371/journal.pone.0003366 18846221 PMC2559863

[B35] Sutton PL Neyra V Hernandez JN Branch OH Plasmodium falciparum and Plasmodium vivax infections in the Peruvian Amazon: propagation of complex, multiple allele-type infections without super-infection Am J Trop Med Hyg 2009 81 950 960 10.4269/ajtmh.2009.09-0132 19996422 PMC3773727

[B36] Joy DA Mu J Jiang H Su X Genetic diversity and population history of Plasmodium falciparum and Plasmodium vivax Parassitologia 2006 48 561 566 17688177

[B37] Silva JC Egan A Friedman R Munro JB Carlton JM Hughes AL Genome sequences reveal divergence times of malaria parasite lineages Parasitology 2011 138 1737 1749 10.1017/S0031182010001575 21118608 PMC3081533

[B38] Carlton JM Escalante AA Neafsey D Volkman SK Comparative evolutionary genomics of human malaria parasites Trends Parasitol 2008 24 545 550 10.1016/j.pt.2008.09.003 18938107

[B39] Cristiano FA Pérez MA Nicholls RS Guerra AP Polymorphism in the Plasmodium vivax msp 3: gene in field samples from Tierralta, Colombia Mem Inst Oswaldo Cruz 2008 103 493 496 10.1590/S0074-02762008000500015 18797765

[B40] Restrepo E Imwong M Rojas W Carmona-Fonseca J Maestre A High genetic polymorphism of relapsing P. vivax isolates in northwest Colombia Acta Trop 2011 119 23 29 10.1016/j.actatropica.2011.03.012 21497586 PMC3485554

[B41] Imwong M Nair S Pukrittayakamee S Sudimack D Williams JT Mayxay M Newton PN Kim JR Nandy A Osorio L Contrasting genetic structure in Plasmodium vivax populations from Asia and South America Int J Parasitol 2007 37 1013 1022 10.1016/j.ijpara.2007.02.010 17442318

[B42] Montoya L Maestre A Carmona J Lopes D Do Rosario V Blair S Plasmodium falciparum: diversity studies of isolates from two Colombian regions with different endemicity Exp Parasitol 2003 104 14 19 10.1016/S0014-4894(03)00112-7 12932754

[B43] Jiménez JN Snounou G Letourneur F Rénia L Vélez ID Muskus CE Near-fixation of a Pfmsp1 block 2 allelic variant in genetically diverse Plasmodium falciparum populations across Western Colombia Acta Trop 2010 114 67 70 10.1016/j.actatropica.2009.12.009 20060375

[B44] Barrera SM Pérez MA Knudson A Nicholls RS Guerra AP Genotypic survey of Plasmodium falciparum based on the msp1, msp2 and glurp genes by multiplex PCR Biomedica 2010 30 530 538 21713358

[B45] Yalcindag E Elguero E Arnathau C Durand P Akiana J Anderson TJ Aubouy A Balloux F Besnard P Bogreau H Carnevale P D’Alessandro U Fontenille D Gamboa D Jombart T Le Mire J Leroy E Maestre A Mayxay M Ménard D Musset L Newton PN Nkoghé D Noya O Ollomo B Rogier C Veron V Wide A Zakeri S Carme B Multiple independent introductions of Plasmodium falciparum in South America Proc Natl Acad Sci U S A 2012 109 511 516 10.1073/pnas.1119058109 22203975 PMC3258587

[B46] Mayor A Aponte JJ Fogg C Saúte F Greenwood B Dgedge M Menendez C Alonso PL The epidemiology of malaria in adults in a rural area of southern Mozambique Malar J 2007 6 3 10.1186/1475-2875-6-3 17233881 PMC1796885

[B47] Guerra-Neira A Rubio JM Royo JR Ortega JC Auñón AS Diaz PB Llanes AB Plasmodium diversity in non-malaria individuals from the Bioko Island in Equatorial Guinea (West Central-Africa) Int J Health Geogr 2006 5 27 10.1186/1476-072X-5-27 16784527 PMC1550388

[B48] Henning L Schellenberg D Smith T Henning D Alonso P Tanner M Mshinda H Beck HP Felger I A prospective study of Plasmodium falciparum multiplicity of infection and morbidity in Tanzanian children Trans R Soc Trop Med Hyg 2004 98 687 694 10.1016/j.trstmh.2004.03.010 15485698

[B49] Pabón A Alvarez G Yánez J Céspedes C Rodríguez Y Restrepo A Blair S Evaluation of ICT malaria immunochromatographic Binax NOW ICT P.f/P.v test for rapid diagnosis of malaria in a Colombian endemic area Biomedica 2007 27 225 235 17713633

[B50] Uscátegui RM Correa AM Carmona-Fonseca J Changes in retinol, hemoglobin and ferritin concentrations in Colombian children with malaria Biomedica 2009 29 270 281 20128352

[B51] Pérez MA Cortés LJ Guerra AP Knudson A Usta C Nicholls RS Efficacy of the amodiaquine+sulfadoxine-pyrimethamine combination and of chloroquine for the treatment of malaria in Córdoba, Colombia, 2006 Biomedica 2008 28 148 159 18645670

[B52] Tangpukdee N Yew HS Krudsood S Punyapradit N Somwong W Looareesuwan S Kano S Wilairatana P Dynamic changes in white blood cell counts in uncomplicated Plasmodium falciparum and P. vivax malaria Parasitol Int 2008 57 490 494 10.1016/j.parint.2008.06.005 18647661

[B53] Prybylski D Khaliq A Fox E Sarwari AR Strickland GT Parasite density and malaria morbidity in the Pakistani Punjab Am J Trop Med Hyg 1999 61 791 801 10586914 10.4269/ajtmh.1999.61.791

[B54] Sutanto I Endawati D Ling LH Laihad F Setiabudy R Baird JK Evaluation of chloroquine therapy for vivax and falciparum malaria in southern Sumatra, western Indonesia Malar J 2010 9 52 10.1186/1475-2875-9-52 20152016 PMC2831905

[B55] Antinori S Galimberti L Milazzo L Corbellino M Biology of human malaria plasmodia including Plasmodium knowlesi Mediterr J Hematol Infect Dis 2012 4 e2012013 22550559 10.4084/MJHID.2012.013 PMC3340990

[B56] Mueller I Galinski MR Baird JK Carlton JM Kochar DK Alonso PL del Portillo HA Key gaps in the knowledge of Plasmodium vivax, a neglected human malaria parasite Lancet Infect Dis 2009 9 555 566 10.1016/S1473-3099(09)70177-X 19695492

[B57] dalla Martha RC Tada MS Ferreira RG da Silva LH Wunderlich G Microsatellite characterization of Plasmodium falciparum from symptomatic and non-symptomatic infections from the Western Amazon reveals the existence of non-symptomatic infection-associated genotypes Mem Inst Oswaldo Cruz 2007 102 293 298 10.1590/S0074-02762007005000044 17568933

[B58] Ariey F Hommel D Le Scanf C Duchemin JB Peneau C Hulin A Sarthou JL Reynes JM Fandeur T Mercereau-Puijalon O Association of severe malaria with a specific Plasmodium falciparum genotype in French Guiana J Infect Dis 2001 184 237 241 10.1086/322012 11424024

[B59] Mockenhaupt FP Bedu-Addo G Junge C Hommerich L Eggelte TA Bienzle U Markers of sulfadoxine-pyrimethamine-resistant Plasmodium falciparum in placenta and circulation of pregnant women Antimicrob Agents Chemother 2007 51 332 334 10.1128/AAC.00856-06 17088491 PMC1797640

[B60] Bouyou-Akotet MK Mawili-Mboumba DP Tchantchou TD Kombila M High prevalence of sulfadoxine/pyrimethamine-resistant alleles of Plasmodium falciparum isolates in pregnant women at the time of introduction of intermittent preventive treatment with sulfadoxine/pyrimethamine in Gabon J Antimicrob Chemother 2010 65 438 441 10.1093/jac/dkp467 20053688

[B61] Niang M Marrama L Ekala MT Alioune G Tall A Ndiaye JL Sarr D Dangou JM Lehesran JY Bouchier C Mercereau-Puijalon O Jambou R Accumulation of CVIET Pfcrt allele of Plasmodium falciparum in placenta of pregnant women living in an urban area of Dakar, Senegal J Antimicrob Chemother 2008 62 921 928 10.1093/jac/dkn299 18682531

[B62] Oster N Rohrbach P Sanchez CP Andrews KT Kammer J Coulibaly B Stieglbauer G Becher H Lanzer M Apparent bias for P. falciparum parasites carrying the wild-type pfcrt allele in the placenta Parasitol Res 2010 106 1065 1070 10.1007/s00436-010-1756-7 20148338

